# Vascular Tumors Result from Adeno-Associated Virus-9 Angiogenic Gene Therapy of Bone Allografts

**DOI:** 10.24238/13221-12-1-192

**Published:** 2020-11-16

**Authors:** Elisa S. Rezaie, Noortje J. Visser, Roman Thaler, Farzaneh Khani, Patricia F. Friedrich, Andrew L. Folpe, David R. Deyle, Alexander Y. Shin, Andre J. van Wijnen, Allen T. Bishop

**Affiliations:** 1 Microvascular Research Laboratory, Department of Orthopedic Surgery - Mayo Clinic; Rochester, Minnesota 55905, USA; 2 Academic Medical Centre, Department of Plastic-Reconstructive and Hand Surgery - Amsterdam University Medical Centre; Amsterdam, 1105AZ, Netherlands

**Keywords:** vascular tumors, adeno-associated, virus-9, angiogenic, gene therapy

## Abstract

**Background:**

Cryopreserved bone allografts are often used to reconstruct segmental bone defects. They are non-viable, which can result in infection, non-union and stress-fractures. We aimed to revascularize allografts in porcine and rat models using vascular endothelial growth factor (VEGF), combined with platelet derived growth factor (PDGF) administered through an adeno-associated viral vector. We report the development of vascular tumors resulting from this treatment.

**Methods:**

In two separate studies, an identical AAV.VEGF.PDGF vector was used to promote angiogenesis in cryopreserved bone allografts. In 8 Yucatan minipigs, a 3.5 cm segmental tibial defect was reconstructed with a matched allograft, revascularized by placement of a transfected arteriovenous (AV) bundle within the medullary canal. In another experiment, cryopreserved femoral bone allografts coated with AAV.VEGF.PDGF were placed across a 10 mm segmental femoral gap in 10 Lewis rats.

**Results:**

Vascular tumors developed in skin and subcutaneous tissues in 5 out of 8 pigs and all of the rats. Histology revealed changes essentially identical to those seen in pyogenic granuloma (lobular capillary hemangioma) in humans. Polymerase chain reaction (PCR) identified the sequence of human VEGF-DNA in all of the sampled tumor tissues.

**Conclusion:**

Recombinant AAV gene therapy used to promote angiogenesis in avascular bone risks the development of vascular cutaneous lesions. Gene therapy using an identical AAV.VEGF.PDGF vector should not be considered clinically until safe use can be demonstrated and.concerns regarding chromosomal integration, dose effect and species differences are addressed.

## Introduction

Vascular endothelial growth factor (VEGF) is the key player in angiogenesis [1] and can be applied experimentally to facilitate angiogenesis. If VEGF is applied outside a narrow therapeutic window, it may result in excessive unorganized vascular proliferation and growth of benign vascular tumors [2, 3, 4]. Combined use of platelet derived growth factor (PDGF) and VEGF has been reported to prevent anomalous angiogenesis and tumor growth by PDGF-directed pericyte recruitment [5, 6]. It has been stated that co-expression of these two factors by a single cell type and gene delivery with a single bi-cistronic vector may represent an effective strategy to support angiogenesis. We applied this experimental approach to generate a neoangiogenic blood supply within cryopreserved bone allografts, with the long-term goal of improving clinical outcomes for patients with large segments of missing bone.

Cryopreserved bone allografts were used to reconstruct segmental bone defects in vivo, a method used frequently in clinical practice. Cryopreserved allografts are readily available clinically from tissue banks, and can be closely matched to any bone defect for excellent initial reconstruction stability. Unlike vascularized autograft bone flaps, allografts remain largely nonviable over time [7]. The freezing process diminishes immunogenicity, with some change in mechanical properties [8, 9]. Without intrinsic vascularity, they are prone to postoperative infection, non-union and late stress fractures [7, 10]. Therefore, it may be desirable to ‘revitalize’ large structural allograft bone segments to improve clinical outcomes.

In this manuscript, we report development of vascular tumors resulting from the use of an adeno-associated bi-cistronic viral vector to promote angiogenesis in two biological models of segmental bone loss reconstructed with cryopreserved allograft. Our findings demonstrate that single-vector expression of PDGF and VEGF may not be a suitable or safe means to promote local therapeutic angiogenesis [5, 6].

## Methods

### Adeno-associated virus

Recombinant adeno-associated virus 9 (AAV9) expressing vascular endothelial growth factor (VEGF-A) and platelet derived growth factor (PDGF) was constructed and produced by the Penn Vector Core at the University of Pennsylvania, using chicken beta actin (CB7) as a promotor, and the woodchuck hepatitis post-transcriptional regulatory element (WPRE) and Chimeric Intron (CI) to increase gene expression. In the AAV cis-plasmid, a rabbit beta-globin polyadenylation sequence is placed after WPRE to ensure formation of a poly(A) tail that aids in transcription termination, export of the mRNA from the nucleus and protein translation, while protecting the mature mRNA from enzymatic degradation in the cytoplasm. The completed vector was identified as AAV9.CB7.CI.GFP.WPRE.rBG with a titer of 5.88×10E13 genome copies/ml (GC/ml), obtained using the droplet digital PCR (ddPCR)-method [11].

### Surgical technique: pigs

A total of 16 female Yucatan mini-pigs (20 kg, 4 months) were SLA-typed and mismatched to donor Yucatan mini-pigs (20 kg, 4 months) from whom they received a cryopreserved segmental tibial bone allograft. The allografts were stored for a minimum of a month at −80°C to reduce immunogenicity prior to surgical implantation.

**Figure 1:**
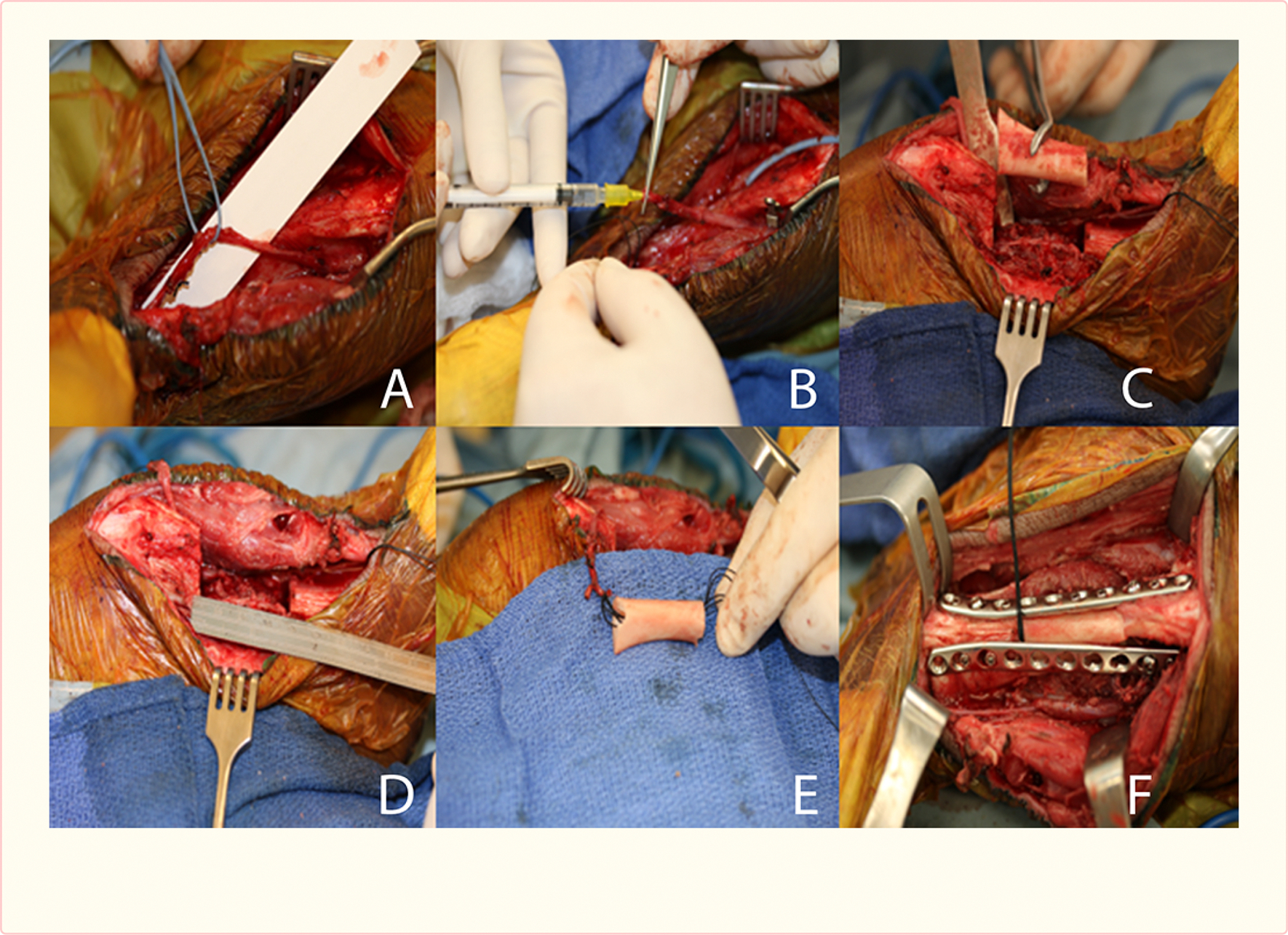
Surgical procedure in pigs respectively showing the AV-bundle (A), injection of AAV.VEGF.PDGF (B), segmental tibial resection (C and D) reconstruction with AV bundle implantation (E), and dual fixation of the allograft (F).

Under sterile conditions a 10 cm incision was made lateral to the anterior ridge of the tibia. The anterior and lateral compartment muscles were reflected from the bone to visualize the cranial tibial artery and vein lying on the interosseous membrane. These vessels were divided at the ankle, ligating the distal vessels stump and occluding flow through the proximal artery temporarily. This arteriovenous (AV) bundle was mobilized towards the knee to permit threading the vessels within a tibial allograft. In 8 of the pigs (VEGF + PDGF group) a bulldog clamp was placed 5 cm proximal to the divided vessel to occlude blood flow. A 24 gauge I.V. catheter (Jelco, Dublin, Ohio) was used to cannulate the artery, followed by delivery of 1×10E13 particles of AAV.VEGF.PDGF suspended in 0.6 ml of PBS, 35 mM NACL and 0.001% Pluronic F68 into the lumen over a period of 20–40 seconds. After injection, the clamp remained in place for an additional 30 minutes to allow transfection of the arterial endothelial cells without arterial inflow. The cannula was then removed and the distal bundle end ligated. The proximal clamp was removed, allowing proximal ingress and egress of blood [Figure 1].

A 3.5 cm segmental defect was next created in the tibial diaphysis immediately distal to the tibial tubercle. It was reconstructed with a cryopreserved allogeneic tibia of matched size and shape, stabilized with dual 9 hole 2.7 mm locking compression plates (Synthes, Monument, CO). A burr was used to create an opening at the proximal and distal allograft coaptation sites, permitting the AV-bundle to be positioned within the allograft. The fascia and skin were closed in layers using 2–0 Vicryl (Ethicon, Somerville NJ) and 3–0 Monocryl cutanous sutures (Ethicon, Somerville NJ). A compressive bandage was applied. A 20-week survival time was planned to study bone healing, angiogenesis and remodeling of the bone.

### Surgical technique: rat femora

A total of 20 male Lewis rats (200–250 gram) served as femoral frozen allogenic bone recipients of femora from 10 female Norway Brown rat donors of identical size.

The 10 donors were anesthetized with isoflurane (3%) and euthanized with Pentobarbital Sodium 390 mg/ml (Vortech Dearborn MI, 200mg/kg intracardiac). Under sterile conditions both femora were removed. A 10 mm diaphyseal segment was prepared by removing all bone marrow, flushed with sterile saline and cryopreserved at −80 °C for a minimum of one month.

**Figure 2:**
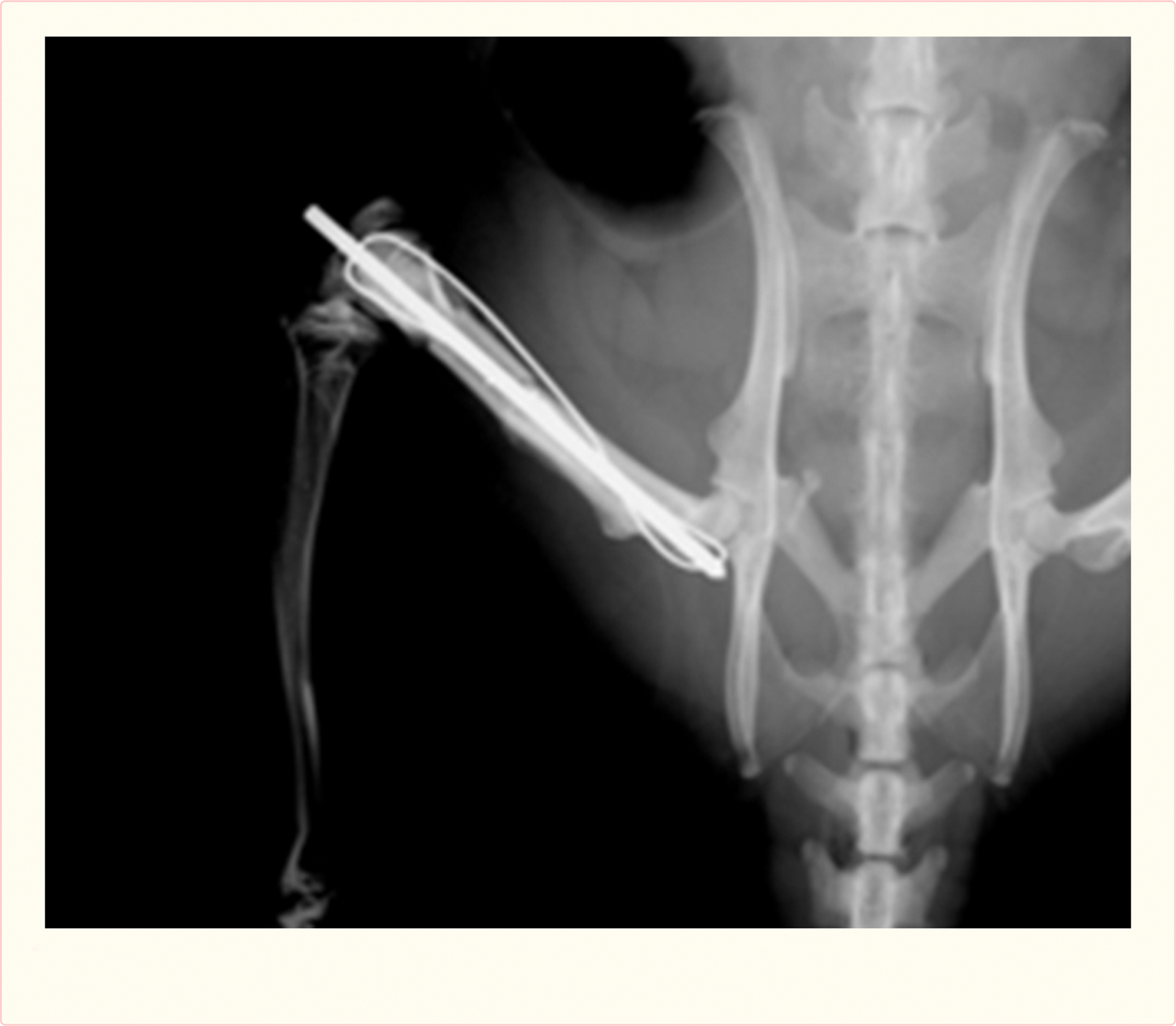
Segmental femoral defect in the rat, reconstructed with intercalary allograft with intramedullary K-wire and tension-band compression wire.

Twenty Lewis rat recipients were used. The rats were anesthetized using ketamine (90 mg/kg intraperitoneal) and xylazine (10 mg/kg intraperitoneal), using additional ketamine intraoperatively (20 mg/kg intraperitoneal) when necessary. A 25 mm skin incision was made over the femur. The lateral vastus muscle and the semimembranosus muscle were separated from the femur. A 10 mm mid-diaphyseal segment was removed and reconstructed with a size-matched Lewis bone allograft. Reconstruction was performed with a 0.045 inch K-wire (Stryker, Kalamazoo, MI) placed as an intramedullary rod with an additional 28 gauge stainless steel tension-band wire providing compression across the reconstruction [Figure 2]. The surface of 10 of the 20 allografts was coated with 1×10E11 particles of the AAV9.VEGF.PDGF). The fascia was closed with 4–0 absorbable sutures (Vicryl, Ethicon, Inc, Somerville, NJ) and the skin with 4–0 nylon monofilament (Ethilon, Ethicon, Inc, Somerville, NJ). Buprenorphine (0.05–0.1 mg/kg subcutaneously) and Baytril (2.5–5 mg/kg subcutaneously) were given postoperatively. Rats were fully weight bearing immediately post-surgery. Survival time was set at 12 weeks, with identical study aims as the swine model study.

In both groups, the unanticipated development of significant numbers of vascular tumors in the AAV groups during the survival period led us to further investigate this issue, forming the subject of this report.

### Polymerase chain reaction

To determine whether the AAV vector was present in the vascular lesions, we examined viral DNA in the affected tissues by monitoring presence of the human VEGF gene. DNA was extracted from vascular lesions in the treated animals, as well as from surrounding unaffected cutaneous and subcutaneous tissues. In addition, we isolated DNA of cutaneous and subcutaneous tissue from 10 unaffected rats and from 8 unaffected pigs that were not treated with any form of gene therapy. The QIAamp DNA mini Kit (Qiagen) was used for DNA extraction from tissue samples. Subsequently, 30 ng of DNA was amplified by conventional PCR using High Fidelity Mastermix (Denville), using 3 minutes of initial denaturation at 95 °C, followed by 35 cycles of 10 s denaturation at 95 °C, 30 seconds annealing at 60°C and 35 seconds extension at 72 °C. The primers used were: 3’ AGGCCAGCACATAGGAGAGA and 5’ ACGCGAGTCTGTGTTTTTG. These specifically amplify the human VEGF gene, generating an amplicon of 247 bp. Finally, the PCR reactions was analyzed on a 2% Agarose gel and imaged with a Gel imaging system (Biorad).

### Histology

Autopsies were performed on all the animals that were treated with AAV9.VEGF.PDGF (10 rats and 8 pigs). The autopsies were performed by an experienced veterinarian that specialized in both pig and rat anatomy. Samples of the tumors, normal surrounding skin and subcutaneous tissue, brain, lungs, heart, kidneys, spleen, pancreas and bone were collected and fixed in formalin 10%. After each autopsy the samples were sent to a veterinary laboratory (Marshfield, Marshfield WI) where they were processed into histology slides. The slides were subsequently examined by an experienced clinical pathologist at our institution.

## Results

**Figure 3:**
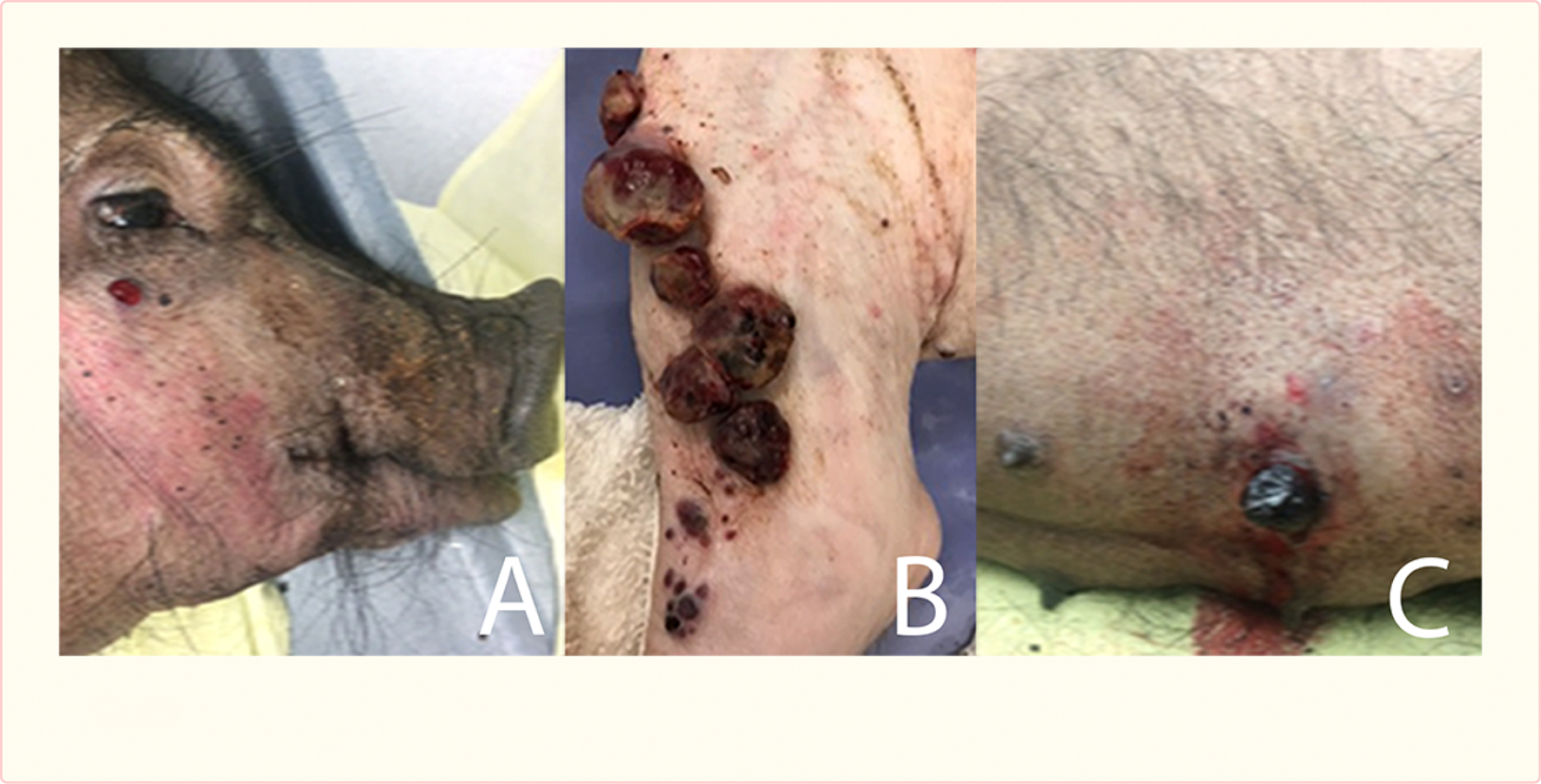
Cutaneous lesions at the, face (A), incision site (B) and trunk (C) in a pig.

**Figure 4:**
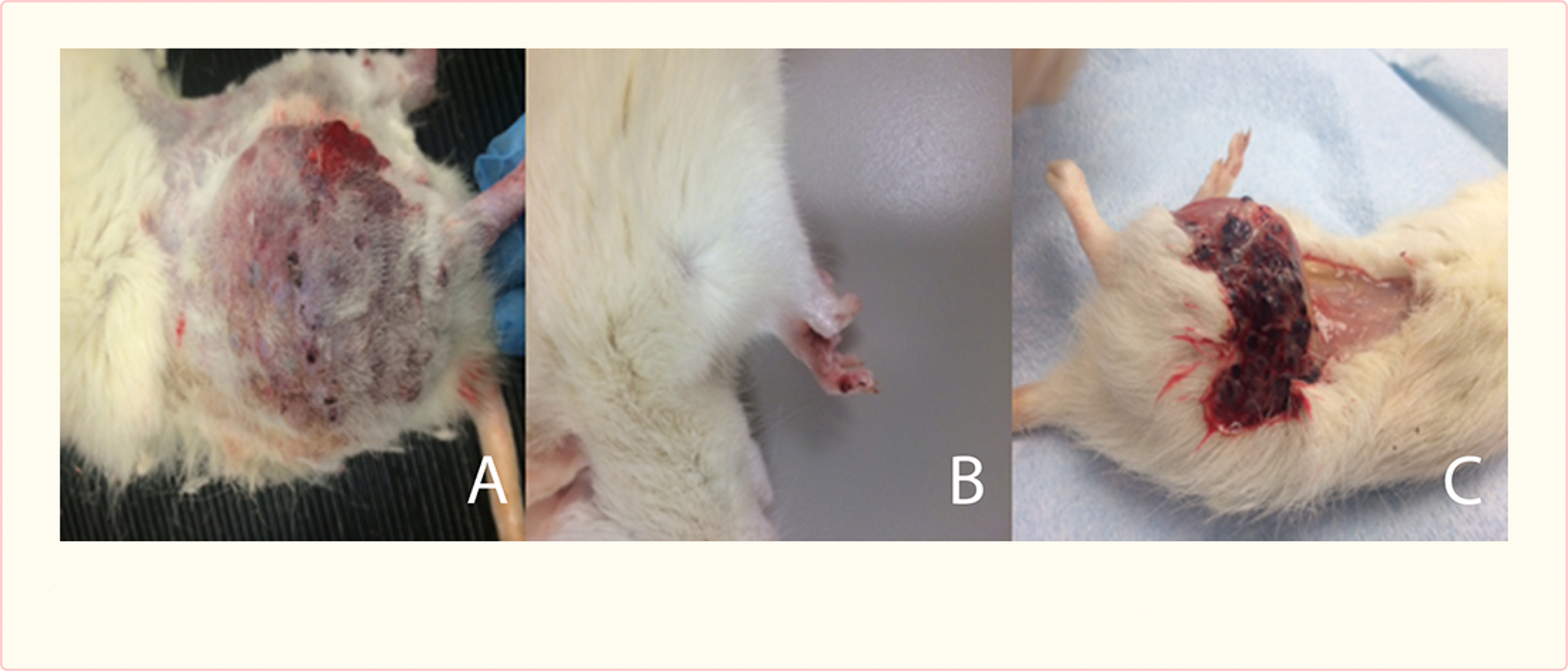
Cutaneous lesions in rats at the incision site (A), and ipsilateral forepaw (B), and beneath the skin at necropsy (C).

During studies of surgical angiogenesis as a means to improve outcomes of cryopreserved bone allografts, we found augmentation of angiogenesis using a bi-cistronic AAV.VEGF.PDGF vector to produce cutaneous and subcutaneous vascular tumors in 5 of 8 pigs and 10 of 10 rats. No tumors were seen before week 10 in pigs, and week 8 in rats. Lesions were found in the operated limb of rats, and additionally in all other limbs, trunk and head of some pigs [Figures 3 and 4]. Autopsies performed in all of the affected animals demonstrated vascular lesions isolated to cutaneous tissues. Gross observation at necropsy and histologic sections failed to demonstrate any lesions in muscle and other deep tissue adjacent to the allograft. Similarly, brain, lung, heart, kidney, spleen and pancreas were unaffected.

### Vascular tumors in AAV-treated pigs

All tumors were cutaneous or subcutaneous in location, forming blood-filled bullae that, once ruptured, resulted in hemorrhage. The animal with the earliest development of tumor (Pig #1) had an initial 1 cm lesion that appeared 10 weeks post-surgery at the incision site. It evolved into 6 lesions 2–5 cm in size and dozens of smaller lesions (1–3 mm) at and distal to the incision site over the next 7 days. At 11 weeks the 6 largest lesions at the incision site were removed surgically to control bleeding. Thereafter, new lesions rapidly appeared in remote locations including the head, other limbs and abdomen at a rate of 2–3 per day. A 2nd surgical procedure at week 12 removed the most severely bleeding lesions from the incision site and abdomen. Two days later, the pig was pale and weakened and was euthanized.

Pig #2 developed the first tumor at 11 weeks post-surgery. The course was much milder, as all lesions developed only on the operated limb, and resulted in minimal bleeding. Over the 20 weeks planned survival time this pig developed 3 lesions at the incision site and another anteriorly. Lesion diameter ranged from 0.5 to 2 cm. The pig’s physical condition remained satisfactory despite the tumors.

Animals # 3 and 4 developed lesions at 14 weeks, again starting at the incision site. Both had extensive development of lesions on the face, ears, neck, all four limbs and abdomen, resulting in significant bleeding. Over the course of the 20 weeks’ survival period, more than 30 cutaneous lesions appeared in each pig, varying from 0.5 cm to 5 cm in diameter. Pig #3 had 3 surgeries performed at 16, 17 and 19 weeks and #4, 4 surgeries performed at 15, 16, 18 and 19 weeks to resect bleeding lesions. During each surgery 2 to 5 lesions that caused significant hemorrhage were removed. New tumors continued to appear thereafter, but the animals’ overall health remained satisfactory, without evidence of pain. As a result, these pigs completed the planned 20 week survival period.

Pig #5 developed a single lesion at 16 weeks, also at the incision site. Its maximum size was 1 cm.

During post-sacrifice autopsy we found additional deep subcutaneous lesions not visible on the surface located at the surgical incision site in pigs #1, #3 and #4. Pigs #1 and #3 had a single subcutaneous lesion less than a centimeter in diameter. Pig #4 had 2 subcutaneous lesions measuring 1 and 5 cm in diameter.

Pig # 6, #7 and #8 received the same treatment with the same dose vector and growth factors and the exact same duration time, but did not develop lesions.

### Vascular tumors in AAV-treated rats

All 10 treated rats developed cutaneous blood-filled bullae, none occurring earlier than 8 weeks post-surgery, all limited to the operated hindlimb. Most were found in proximity to the surgical site. Three animals developed a single lesion on the ipsilateral hindlimb paw. Lesions grew substantially in size and in number in all rats [Figure 4]. None resulted in substantial hemorrhage, and none were euthanized prior to the 12 week survival period. In 2 animals, difficulty during walking was observed, likely due to the weight of the mass of tumors.

### Histology

Histologic examination was performed on many of the tumors from the both pigs and rats. Sampling of multiple lesions from the same animal typically showed lesions in different apparent stages of evolution, such that some inferences could be drawn about their morphological progression over time. The lesions began with capillary and myofibroblastic proliferation, followed by vascular thrombosis and reorganization, and eventual fibrous obliteration of the neoangiogenic vascular structures.

**Figure 5:**
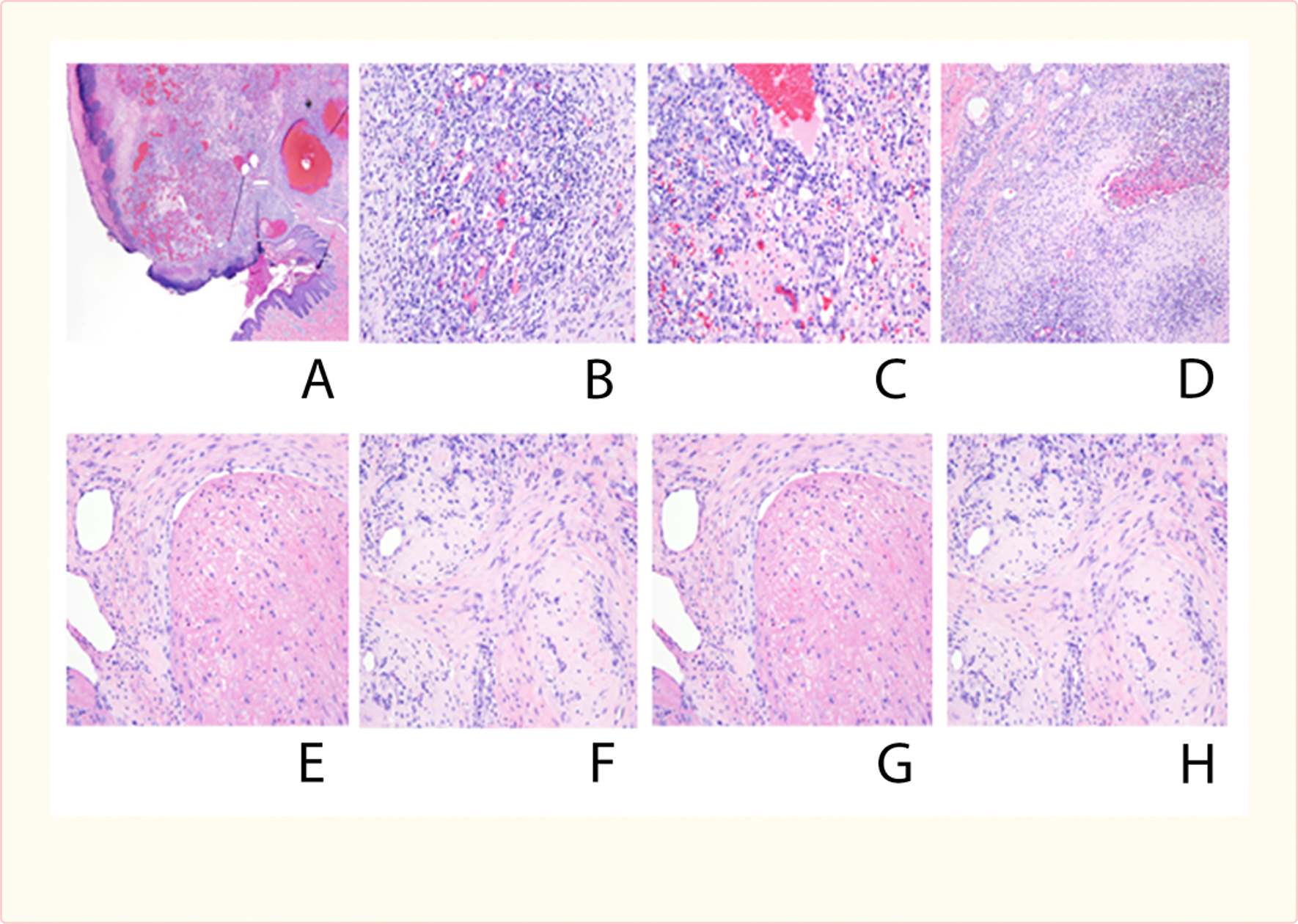
Pig histology showing evolution of an exophytic pattern of growth with surface ulceration and formation of an epidermal collarette (A), exuberant proliferation of small capillary-sized blood vessels, lined by a single layer of slightly protuberant but normochromatic endothelial cells (B), inflammatory cell infiltrate (C), intravascular thrombi (D), large, blood-filled ectatic spaces, surrounded by more cellular foci of capillary proliferation (E), thrombosed vascular channels undergoing fibrous obliteration (F), residual lumina, with surrounding fibroblastic/myofibroblastic proliferation and collagen deposition (G), and fibrous scars, representing clusters of entirely reorganized vascular channels (H).

At scanning magnification, the lesions typically showed an exophytic pattern of growth with surface ulceration and formation of an epidermal collaret, changes essentially identical to those seen in pyogenic granuloma (lobular capillary hemangioma) in humans [Figure 5a]. Higher power magnification disclosed an exuberant proliferation of small capillary-sized blood vessels, lined by a single layer of slightly protuberant but normochromatic endothelial cells [Figure 5b]. A mixed inflammatory cell infiltrate consisting of small lymphocytes, neutrophils, and occasional eosinophils and plasma cells was present [Figure 5c]. In rare cases, morphologically normal mitotic figures could be identified in endothelial cells and in surrounding myofibroblasts.

Lesions in an apparently later stage of evolution were most notable for the presence of intravascular thrombi, with varying degrees of organization and endothelial ingrowth [Figure 5d]. These thrombosed vascular structures were often distended, creating large, blood-filled ectatic spaces, surrounded by more cellular foci of capillary proliferation [Figure 5e]. These thrombosed vascular channels appeared eventually to undergo fibrous obliteration [Figure 5f], resulting in near-total occlusion and leaving only small, residual lumina, with surrounding fibroblastic/myofibroblastic proliferation and collagen deposition [Figure 5g]. Very late-stage lesions consisted of multiple fibrous scars, representing clusters of entirely reorganized vascular channels [Figure 5h].

**Figure 6:**
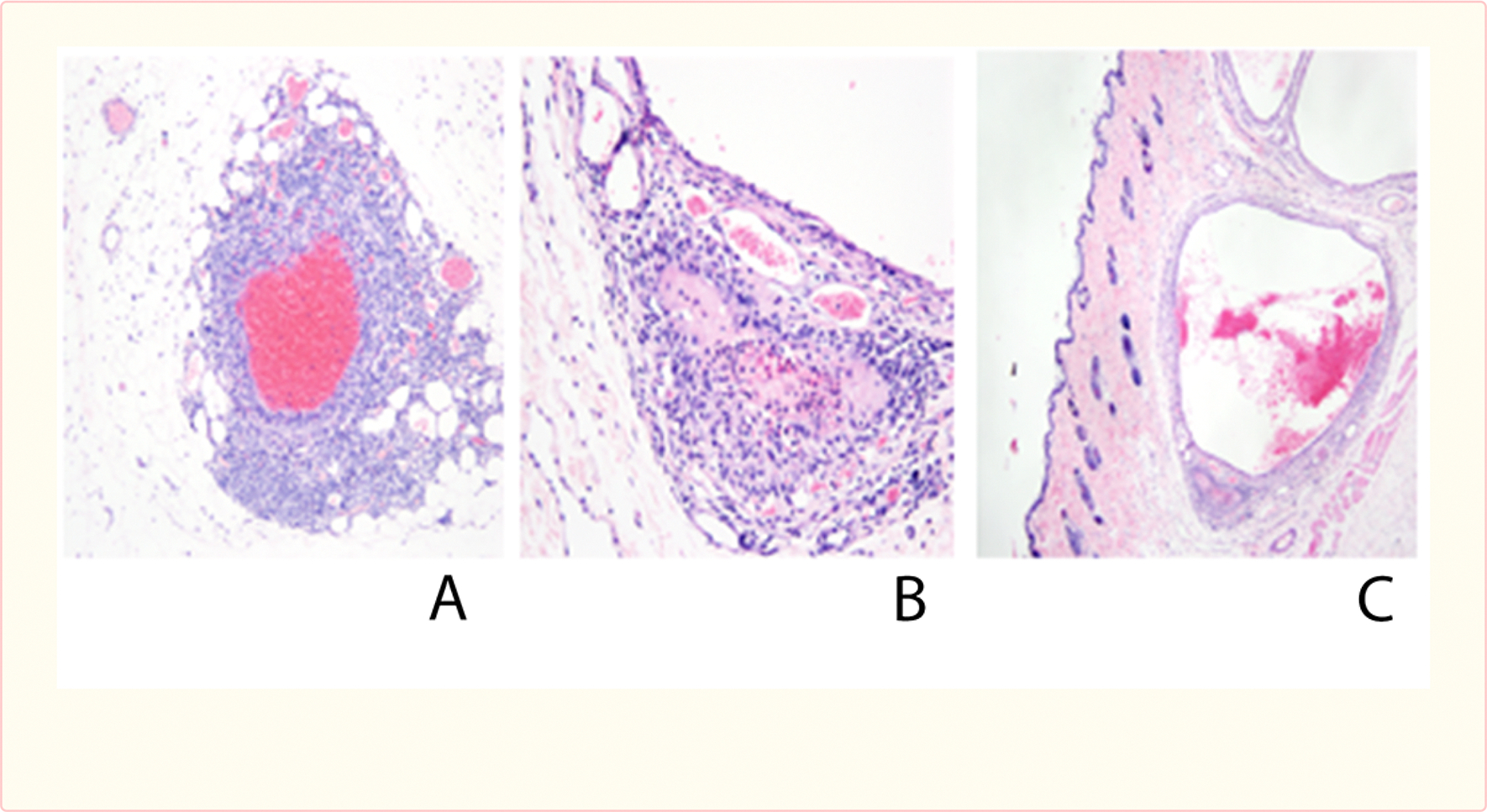
Similar changes were seen in section of skin from the rats, with foci of cellular capillary proliferation (A), thrombosis (B), and ectasia (C).

Similar changes were seen in section of skin from [Figure 6a], thrombosis [Figure 6b], and ectasia the rats, with foci of cellular capillary proliferation [Figure 6c].

### Polymerase Chain Reaction

**Figure 7:**
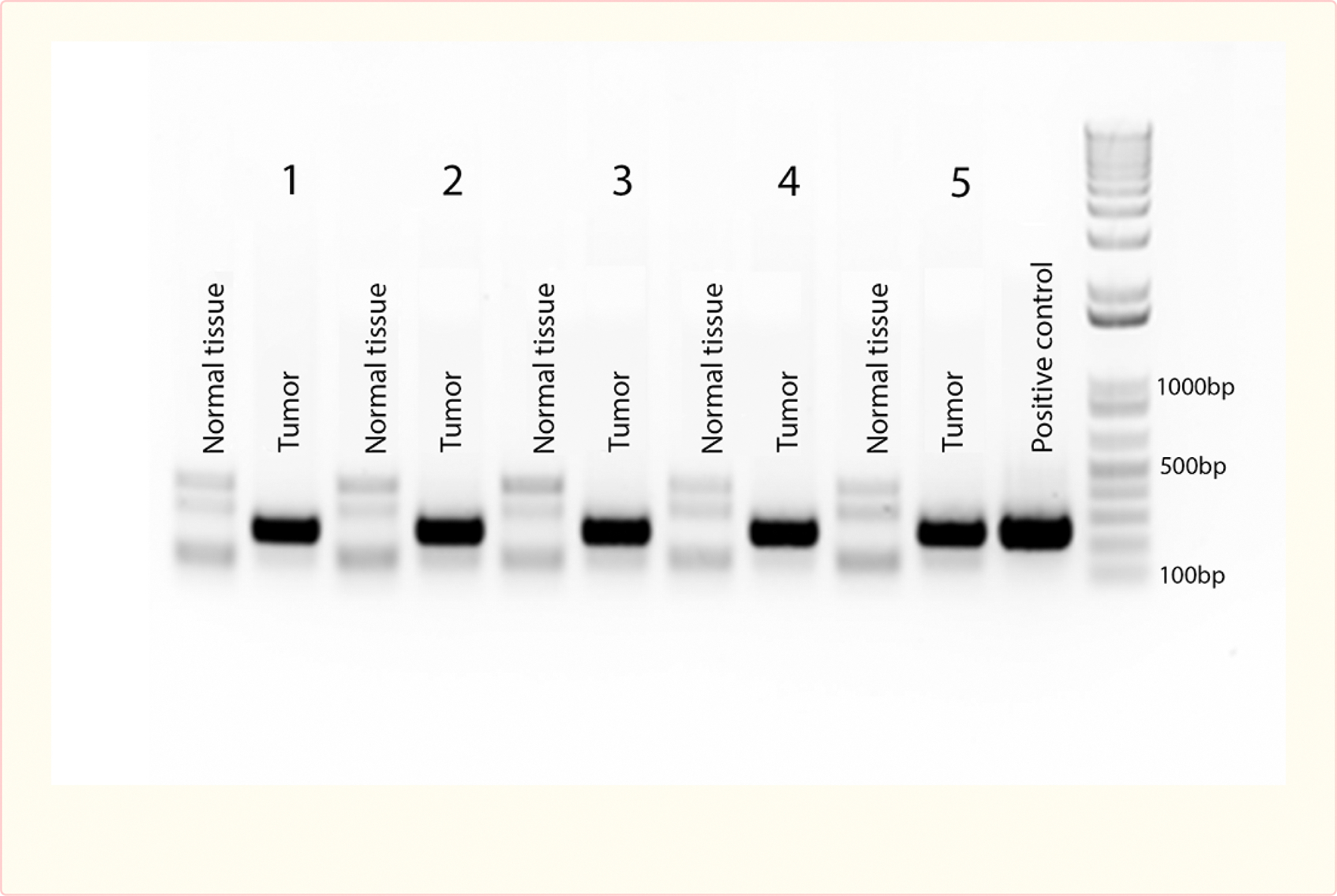
Agarose gel electrophoresis of PCR amplifications on DNA samples from pig tumors and adjacent normal tissue to identify the human VEGF gene. The adeno-associatred virus AAV.VEGF.PDGF was used as the positive control. Tumor and normal tissues are from each of the five pigs that developed tumors. The last bar is the DNA ladder.

**Figure 8:**
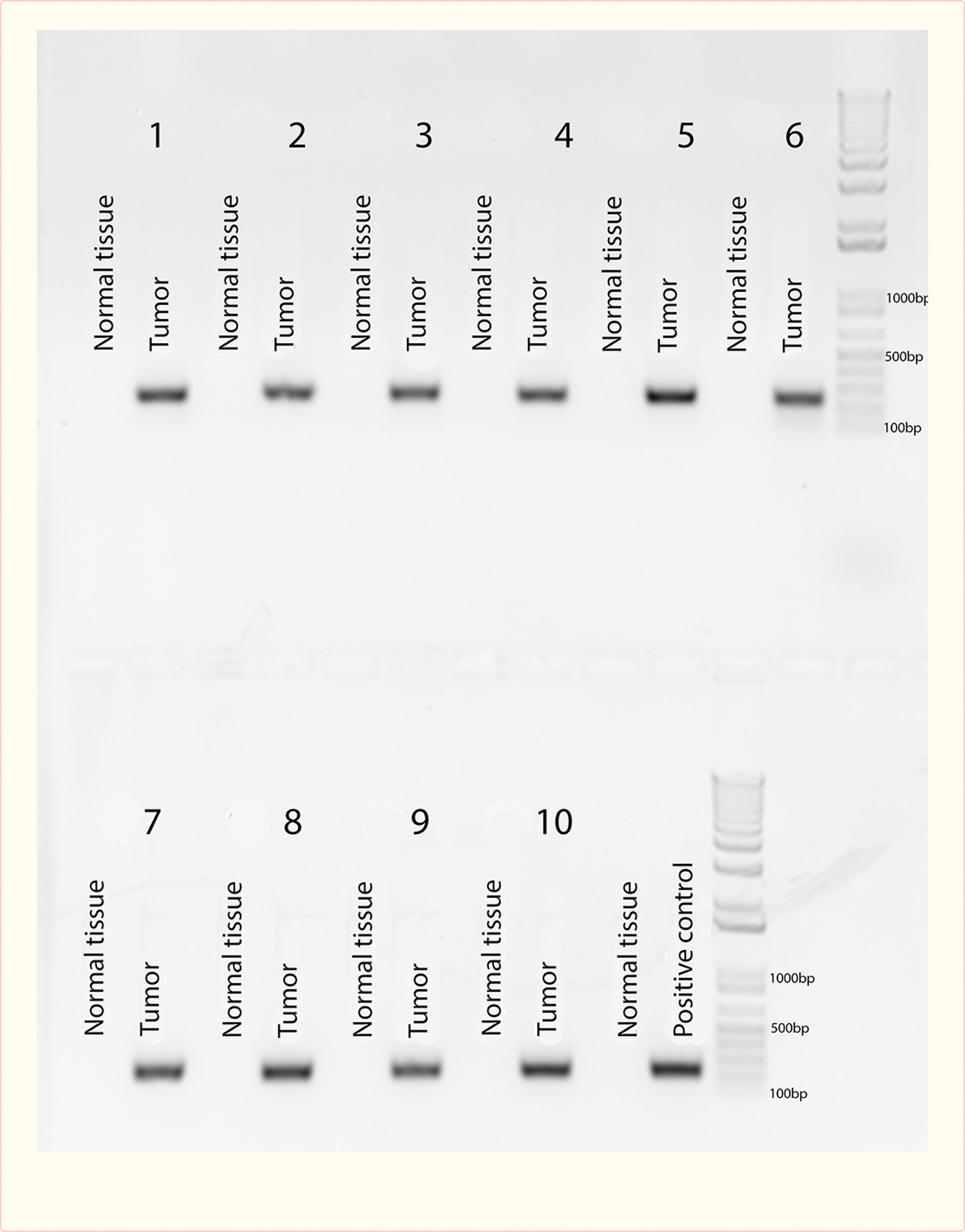
Agarose gel electrophoresis following PCR human VEGF amplification of a tumor and normal tissue from each of the ten rats, with the same positive control as Figure 7.

Polymerase chain reaction (PCR) was performed on representative lesions from all AAV9.VEGF.PDGF treated rats and pigs, as well as on uninvolved tissue that appeared normal upon visual inspection. The viral vector itself served as a positive control, and cutaneous tissue from an untreated rat and an untreated pig served as a negative controls. We identified viral DNA representing an 247 bp long fragment amplified from the 1239 bp long sequence of the human VEGF gene in the positive control and in all of the tumor tissues, but not in healthy adjacent tissue of the experimental animals or negative controls which only show the generation of unspecific bands [Figures 7 and 8]. Treatment of the samples with RNase A during DNA extraction allowed us to avoid false positive results by possible hVEGF RNA-molecules in the samples. These results indicate that human hVEGF DNA-sequences were found specifically in tumors at sites distant from the original site of viral delivery suggesting chromosomal integration of the AAV9.VEGF.PDGF vector.

## Discussion

In previous rodent and canine studies from our laboratory in which growth factors were used to promote bone allograft revascularization, no vascular tumors developed [12, 13, 14, 15]. We have used a variety of methods to provide local VEGF delivery, including encapsulation of growth factors in biodegradable microspheres placed within the allograft, gene therapy with a replication-deficient recombinant adenoviral vector, or sustained local delivery by osmotic pump. When microspheres are used, there is an initial burst of growth factor release, followed by a sustained zero-order kinetic release for 28 days [13, 16, 17, 18, 15]. In addition, VEGF has been directly administered as a single dose of 1×109 units per milliliter [12], or by continuous infusion using an osmotic pump for 3 days [14]. None of these three non-viral delivery methods (i.e., microspheres, direct injection, or pump) generated detectable lesions. Furthermore, no tumors were seen in AV-bundles in which endothelial cells were transfected with a replication-deficient adenoviral vector to produce hVEGF. Adenovirus mediated gene expression has a more limited expression time than AAV, ranging from a 2 weeks to a few months [19]. Our current studies were performed with much higher viral titer (1×109 pfu/ml) [12] and an adeno-associated virus. The higher viral titers, the longer period of gene expression provided by AAV vectors (>1 year), and the use of a both VEGF and PDGF are potentially important differences from our prior work [20, 21, 22].

There are several other reports on formation of angioma-like tumors after AAV gene-therapy [2, 23, 3, 24, 4, 25]. Karvinen and colleagues [23] studied the effects of long-term VEGF expression in rabbit hindlimbs administered through AAV. They injected a dose of 1×1011 virus particles into the rabbit semimembranosus hindlimb muscle, with a maximal survival time of 12 months. This study had positive outcomes (e.g., increased local perfusion, capillary enlargement and sprouting) indicating that AAV-VEGF has significant potential for therapeutic use. However, the authors also observed aberrant angiogenesis suggesting that the risk of these side effects may prevent clinical use of currently available AAV vectors.

Banfi and colleagues [5] found co-delivery of PDGF and VEGF to eliminate aberrant angiogenesis irrespective of VEGF levels, especially when both were administered through a bi-cistronic vector. They used an adenovirus, with a titer of 1X108 particles injected into posterior auricular muscles and tibialis anterior muscles of mice. When VEGF was administered alone, it induced abundant aberrant vascular structures after 2 weeks, which later evolved into large angiomas, but homogenous normal capillaries were formed when PDFG and VEGF were co-expressed from the same bi-cistronic vector. Similar findings were obtained when bi-cistronic myoblasts were used in a hindlimb ischemia model. The study has some differences from our report. Analysis of angiogenesis was limited to the transfected muscle tissue, and the survival time was a short 2 weeks. Our study, in contrast, found a large amount of abnormal vessel formation in the rat despite bi-cistronic delivery. It may well be that the animal model, their limited survival time, the considerably lower dose and/ or the difference in vectors used (myoblast and adenovirus versus adeno-associated virus) account for the differences when compared to our study.

Kupatt and co-workers [26] studied gene therapy to improve myocardial perfusion, comparing VEGF alone to VEGF + PDGF in pig myocardium. They found that VEGF, when combined with a PDGF-B in an AAV bi-cistronic vector delivered by a dose of 4 × 1012virus particles resulted in an increase of myocardial perfusion and function. Transfection with VEGF alone did not improve myocardial perfusion or function, although angiogenesis was efficiently induced. No aberrant angiogenesis was seen in either group, although the longest survival time was 56 days. As we did not see aberrant angiogensis until 70 days at the earliest in the same animal model, it is possible a longer survival period would have demonstrated vascular lesions.

Gianni-Barrera et al. [6] also looked at in vivo expression of VEGF and PDGF through myoblast delivery in severe combined immunodeficiency (SCID) mouse ear and leg muscles. Their longest survival time was 4 months. Bi-cistronic administration of VEGF and PDGF delivered by myoblasts promoted safe angiogenesis for the duration of the survival period.

We selected a recombinant AAV-9 virus as our vector based upon our literature review. AAV-9 has the widest diversity of target tissues and it has shown to be a potent vector for gene delivery to both dividing and non-dividing cells, with long-term expression lasting for years [27]. We aimed to deliver our genes to a single desired location with the techniques selected. AAV9 has shown to bind to galactose on cell surface membranes, making it readily transducible in multiple tissues. Although low concentrations of AAV may be desirable to limit spread, a large number of rAAVs optimizes transfection. We chose a high concentration and dose of rAAV for this reason. rAAV has been described to be a safe vector with no pathogenic features such as active integration into the genome [28, 29]. The viral genomes are expected to remain episomal after transduction, as the proteins needed for integration are absent, unlike the wild-type virus [30].

Our finding of human VEGF in vascular lesions calls this into question, as cell division within the lesions should have serially diluted the concentration of the delivered gene [31]. While we did not formally establish whether the AAV vector is integrated, the detection of AAV vector sequences in the tumor is suggestive of vector genome integration, replicated passively during cell division. Our findings provide a cautionary observation requiring reconsideration of the safety of AAV vectors for human gene therapy, certainly for when expressing the angiogeneic growth factor VEGF.

These data also demonstrate that co-expression of PDGF and VEGF using a bi-cistronic recombinant AAV vector does not in fact prevent aberrant angiogenesis. Instead, the development of angioma-like tumors caused sufficient bleeding to require repeated surgical excision and even euthanasia in some Yucatan minipigs, and occurred uniformly in all treated rats.

The sustained expression of human VEGF seen weeks after AAV vector tissue transfection in both rats and pigs, desirable for its angiogenic effect, unfortunately caused highly morbid benign vascular tumor proliferation with cutaneous hemorrhage. These effects were less severe in rats, in whom the virus was coated on bone rather than delivered into an arterial lumen. Both methods of delivery and species differences may be factors in this observation. It remains to be established as to why the lesions were limited to skin and subcutaneous tissue.

## Conclusion

In conclusion, recombinant AAV gene therapy with VEGF and PDGF, when used to promote angiogenesis in avascular bone resulted in development of benign vascular tumors in cutaneous tissues of rats and pigs. Co-expression of VEGF and PDGF did not prevent the formation of aberrant vascular tumors as others have reported, and viral DNA present in all tumors suggests possible chromosomal integration of the AAV viral genome. Tumors did not appear until 8 to 10 weeks after viral delivery, and continued to develop over the survival period. Further investigation regarding possible chromosomal integration as well as the role of dose and species-specific effects is required.
